# Correlated fluorescence microscopy and multi-ion beam secondary ion mass spectrometry imaging reveals phosphatidylethanolamine increases in the membrane of cancer cells over-expressing the molecular chaperone subunit CCTδ

**DOI:** 10.1007/s00216-020-03013-9

**Published:** 2020-10-31

**Authors:** John S. Fletcher, Sanna Sämfors, Josefine Vallin, Andreas Svanström, Julie Grantham

**Affiliations:** 1 Department of Chemistry and Molecular Biology, University of Gothenburg, 412 96 Gothenburg, Sweden; 2 Present Address: Department of Chemistry and Chemical Engineering, Chalmers University of Technology, 412 96 Gothenburg, Sweden; 3 Present Address: Department of Laboratory Medicine, Institute of Biomedicine, Sahlgrenska Academy, Sahlgrenska Center for Cancer Research, University of Gothenburg, 413 90 Gothenburg, Sweden

**Keywords:** ToF-SIMS, Fluorescence microscopy, Chaperonin, Lipids, Phosphatidylethanolamine, Membrane curvature

## Abstract

Changes in the membrane composition of sub-populations of cells can influence different properties with importance to tumour growth, metastasis and treatment efficacy. In this study, we use correlated fluorescence microscopy and ToF-SIMS with C_60_^+^ and (CO_2_)_6k_^+^ ion beams to identify and characterise sub-populations of cells based on successful transfection leading to over-expression of CCTδ, a component of the multi-subunit molecular chaperone named chaperonin-containing tailless complex polypeptide 1 (CCT). CCT has been linked to increased cell growth and proliferation and is known to affect cell morphology but corresponding changes in lipid composition of the membrane have not been measured until now. Multivariate analysis of the surface mass spectra from single cells, focused on the intact lipid ions, indicates an enrichment of phosphatidylethanolamine species in the transfected cells. While the lipid changes in this case are driven by the structural changes in the protein cytoskeleton, the consequence of phosphatidylethanolamine enrichment may have additional implications in cancer such as increased membrane fluidity, increased motility and an ability to adapt to a depletion of unsaturated lipids during cancer cell proliferation. This study demonstrates a successful fluorescence microscopy-guided cell by cell membrane lipid analysis with broad application to biological investigation.

Graphical abstract

**Electronic supplementary material:**

The online version contains supplementary material available at 10.1007/s00216-020-03013-9.

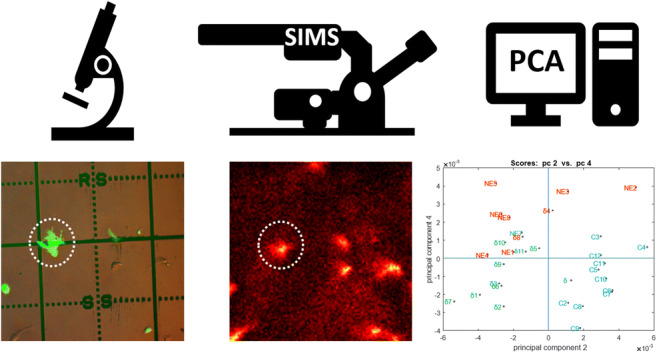

## Introduction

While different organs within an organism comprise a range of different cell types with specific function, there is increasing interest in the diversity within the population of each cell type. Phenotypic changes, often with a change in metabolic/lipid profile, can occur within a population of cells leading to disfunction or, in the case of cancer, altering the potential response to treatment or the ability of the cell to infiltrate surrounding tissue leading to metastasis [1].

The plasma membrane, together with the underlying membrane-associated cytoskeletal networks, acts as a sensor for responding to extracellular cues, for example from growth factors. Numerous processes such as endocytosis, exocytosis and filopodia growth involve the dynamic formation of high curvature regions within the plasma membrane at specific locations. Curvature of the plasma membrane is a consequence of lipid composition, occurrence of curvature-inducing lipid-binding proteins, protein insertion into the lipid bilayer and cytoskeleton-induced tension [2].

Hence, there is a requirement for analytical approaches that can provide detailed lipid information*,* in situ, on a cellular scale with surface sensitivity. Secondary ion mass spectrometry (SIMS) is one potential candidate but has previously been limited in the amount of intact lipid signals, often detecting smaller fragments of the lipid molecules that are less biologically specific. SIMS, using a focused ion beam to eject atoms and molecules from a sample surface, provides a unique means of probing the chemical composition of the surface of cells. Ultimately, the ion beams can be focused to 10’s of nanometres but this comes with a trade off in terms of molecular sensitivity and so imaging biomolecules is often limited to around 1 μm and often only for small molecules or fragments of molecules with very high abundance and/or ionisation probability [3–7].

Gas cluster ion beams (GCIBs) have greatly increased the ability to generate SIMS signals from intact lipids and have been employed in a range of biological studies, from cancer tissue imaging to bacterial membrane analysis [8–17].

**Fig. 1:**
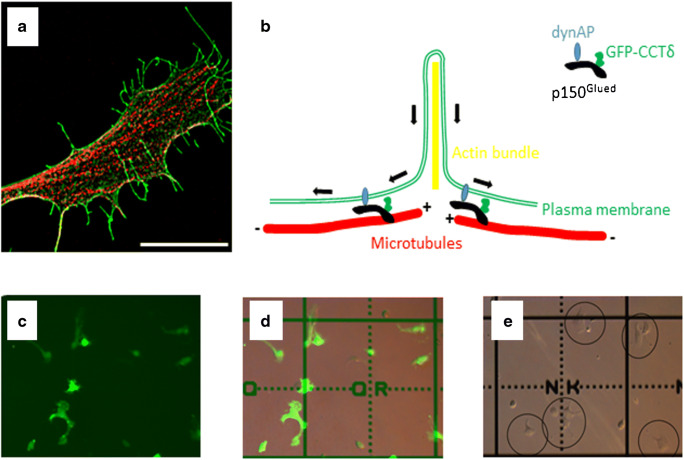
Induction of plasma membrane tubulation via GFP-CCTδ expression for single-cell analysis. A B16F1 mouse melanoma cell transfected with GFP-CCTδ (green) and then immunostained for α-tubulin (red) visualized by structured illumination microscopy is shown in **a**. The size bar corresponds to 10 μm. **b** Cartoon illustrating the inward movement of the plasma membrane mediated by the transmembrane protein dynAP, together with p150^Glued^ and GFP-CCTδ upon expression of GFP-CCTδ, according to Echbarthi et al. [21]. **c** to **e** Cells were plated on metal-coated, finder grid-marked, slides and transfected with GFP-CCTδ (**c**, GFP channel) and (**d**, GFP channel merged with transmitted light) or with transfection reagents and no DNA as a control (**e**, transmitted light). Examples of cells in (**e**) are circled for clarity. In this format, a single cell can be selected and a mass spectrum is generated from that cell for statistical analysis

Here, ToF-SIMS is used for investigating changes in membrane lipid composition that could influence membrane curvature. The cell phenotype induced by over-expression of the CCTδ protein in cultured mammalian cells is utilised. CCTδ is a component of the multi-subunit molecular chaperone named chaperonin-containing tailless complex polypeptide 1 (CCT) or tailless complex polypeptide 1 ring complex (TRiC). The CCT oligomer is well-characterised as an essential molecular chaperone for the folding of actin and tubulin (and a range of other, less abundant proteins), while some of its subunits, when monomeric, possess additional functions [18]. When CCT subunits are fused to GFP at their N-termini, their incorporation into the CCT oligomer is hindered providing a means to study the cellular functions of the CCT subunits when monomeric [19, 20]. In cultured mammalian cells, expression of GFP-CCTδ, which accumulates close to the plasma membrane, results in the inward movement of the plasma membrane, resulting in numerous long, thin fibres forming around bundles of actin filaments [19]. For this phenotype to form the transmembrane protein dynAP, p150^Glued^ (a component of the dynactin complex) and microtubules are required, suggesting that CCTδ: p150^Glued^ interactions may provide connections between the plasma membrane (via dynAP) and microtubules [21]. Thus, these thin, protruding, membrane structures are formed by mechanical forces exerted most probably by dynein:dynactin-mediated transport along microtubules towards the centre of the cell/microtubule minus ends. An image of a B16F1 mouse melanoma cell expressing GFP-CCTδ and stained for α-tubulin to visualize microtubules is shown in Fig. 1 a and a cartoon summary of the mechanism of the phenotype generation according to Echbarthi et al. [21] is shown in Fig. 1 b.

There are numerous potential connections between CCT and cancer cell biology [18]. In particular, the levels of the CCT subunits have been linked to cell growth and more recently the CCTβ subunit has been associated with invasion and proliferation [22, 23]. The CCTδ subunit is one of the CCT subunits shown to have increased expression in an in vivo invasion assay [24]. Also, differences in cell migration in wound healing assays, depending on expression levels of GFP-CCTδ, have been observed in comparison to cells expressing GFP-CCTβ or mutants of GFP-CCTδ [21]. However, to date, the effect of CCT subunit levels on membrane composition is not known.

In this study, we use a correlative fluorescent microscopy, SIMS imaging approach where both C_60_^+^ and (CO_2_)_6k_^+^ ion beams are used. This allowed specific cells showing a phenotypic change following successful plasmid transfection to be selected. Changes in the lipid profile of these cells compared with control cells were identified, particularly an increase in phosphatidylethanolamines (PEs). Such lipid changes are implicated in the formation of high curvature structures on the cell surface.

## Methods

### Preparation of gold grid microscope slides

Grid microscope slides with a cell finder pattern made from chrome etched onto soda lime glass were purchased from JD Photo Data. The slide had ten squares of 10 × 10 mm^2^ where each square was further divided into 625 squares of 0.4 × 0.4 mm^2^. The 625 squares were coded with two letters of the alphabet making each square unique. The slide was cut into 1 × 1 cm^2^ pieces (one piece containing the 625 squares) using a diamond glass cutter. In order to make the slide pieces conductive, they were coated with a 3-nm-thick layer of titanium followed by a 12-nm-thick layer of gold using an electron beam thin film evaporator (Kurt J. Lesker). Prior to cell seeding, the pieces were sterilized in 70% ethanol.

### Cell culture

The mouse melanoma cell line B16F1 (provided by Prof Roger Karlsson, The Wenner-Gren Institute, Stockholm University) was selected due to a high transfection efficiency and suitability to imaging. Cells were maintained in DMEM (GIBCO Life Technologies) supplemented with 10% FBS (Invitrogen), 100 U/ml Penicillin-Streptomycin (GIBCO Life Technologies) and 2.5 μg/ml Plasmocin (InvivoGen) in a humidified atmosphere of 5% CO_2_ at 37 °C. Two days prior to transfection, cells were plated at a density of 2 × 10^4^ cells/well in a standard six-well cell culture plate. Transfection of 2 μg/well DNA was performed using Lipofectamine 2000 transfection reaction (Invitrogen) according to the manufacturer’s instructions and OptiMEM media (GIBCO Life Technologies). The plasmid expressing GFP-CCTδ is described in Spiess et al. [19]. One day post transfection, cells were harvested using 1 mM EDTA/PBS and re-plated onto metal-coated, finder grid-marked, slides pre-coated with laminin (Sigma) at an appropriate cell concentration to give a cell density where the majority of cells had no contact with other cells. The cells were then incubated in a humidified atmosphere of 5% CO_2_ at 37 °C for 2.5 h to allow for attachment to the surface of the silicon wafer.

### Structured illumination microscopy

For structured illumination microscopy, GFP-CCTδ transfected B16F1 cells plated on laminin-coated glass coverslips were fixed and stained with an antibody to α-tubulin/anti-mouse secondary antibody conjugated to Alexafluor594 as described previously [21]. Images were taken using a Zeiss ELYRA PS.1 LSM780 system with Zeiss Zen Software.

### Wide-field fluorescence microscopy

Prior to sample preparation for mass spectrometry, wide-field images were taken of the live cells with a Zeiss Axioplan microscope using AxioVision software. In this case, no chemical treatment or additional labelling of the cells was required as the transfected cells expressed GFP.

### ToF-SIMS

ToF-SIMS analysis was performed using a J105 instrument (Ionoptika Ltd, UK). The instrument has been described in detail elsewhere [25]. Briefly, the J105 differs from most ToF-SIMS instruments in the removal of the requirement for short pulsing of the primary ion beam. Instead, the secondary ion stream is bunched to a time focus at the entrance to a reflectron ToF mass analyser. The instrument is equipped with 40 kV C_60_^+^ and gas cluster ion beam (GCIB) systems. The GCIB used in this study was a (CO_2_)_6k_^+^ beam that was selected using a Wien filter and the size verified by time-of-flight measurement from a pulser in the ion gun to the sample surface.

The SIMS analysis was performed on the same cells imaged using the wide-field fluorescence microscopy.

GCIB imaging was performed using a pixel size of 6.25 μm and the C_60_^+^ imaging using a pixel size of 1.56 μm. The target currents as measured in a Faraday cup were 20 pA and 9 pA respectively. The primary ion dose density of the GCIB analysis was 2.56 × 10^12^ ions/cm^2^ for each ion polarity. While the exact erosion rate of these cells is not known, this would be equivalent to approximately 12 nm erosion on Irganox 1010, a common organic reference material for ToF-SIMS [8].

### Cell selection

Fluorescent images obtained by wide-field microscopy were used for identifying the transfected cells and only transfected cells expressing an obvious phenotype change were selected for comparison with control cells. In the GCIB analysis, the cells were visualised using the peak at *m/z* 184.07 corresponding to the phosphatidylcholine head group for positive ion mode and the *m/z* 885.5 corresponding to PI(38:4) for negative ion mode. To identify the transfected cells in the ion images, the total ion image from the high-resolution C_60_ analysis was used since the grids were visible in the ion image allowing comparison to the fluorescent microscope image. GCIB analysis was performed on the exact same spot on the sample as the C_60_, which allowed for identification of the same cells in the two different ion images by a manual overlay of the images.

### Principal components analysis

Principal components analysis (PCA) was performed on the single-cell spectra. PCA is used to identify differences/variance in sample data (spectra) where the first principal component (PC1) captures the most variance PC2 the second largest variance and so on. The results of the analysis are in the form of scores and loadings. Plotting the scores can provide a visual indication as to which samples are different/similar and the variables (*m/z* values) responsible for these differences are provided in the loadings. Loadings can be plotted on a variable (*m/z*) axis that illustrates positive loading and negative loading peaks. A sample that scores positive would have a relatively higher signal from the positive loading peaks than the negative loading peaks and vice versa for negative scoring samples. Data was imported into Matlab2019b and the mass/time resolution down sampled from 1 to 30 ns to reduce noise and account for any possible drift in mass calibration. The mass range was truncated to remove data points above *m/z* 1000 where no clear peaks were observed and remove lower mass species arising from intense fragment ions such as the fatty acids (RCO^−^ ions at *m/z* 200–250) in negative ion mode. Removal of the lower mass range also reduces contribution from the substrate and small salt cluster ions. The signal was then normalised to the total ion signal for the selected *m/z* range and the data was mean centred before PCA was performed using the NIPLS algorithm.

## Results and discussion

SIMS, as with many desorption ionisation mass spectrometry techniques, can be prone to sample charging when insulators are analysed and so samples are often analysed on electrically conducting substrates. While metal-coated (typically indium tin oxide) glass microscope slides are available, more specialised slides incorporating grid markings, etc., are not normally supplied with a metal coating. Hence, a transparent metal coating was applied to the grid microscope slides by evaporating first Ti, followed by Au. Inspection of the cells by fluorescent microscopy indicated the successful transfection of approximately 70% of the cells based on the fluorescent signal visible from the expression of GFP-CCTδ (160 cells were counted from a total of four fields of view). Furthermore, fluorescent cells clearly displayed the phenotypic changes expected by the expression of GFP-CCTδ, namely extensive formation of tubular structures on the cell surface. Hence, the use of the gold-coated grid slides had no adverse effect on either the cell growth, transfection of or the optical microscopy steps in the experimental workflow. The grid references on the microscope slides allowed target areas to be selected for ToF-SIMS analysis as shown in Fig. 1 d and e.

**Fig. 2:**
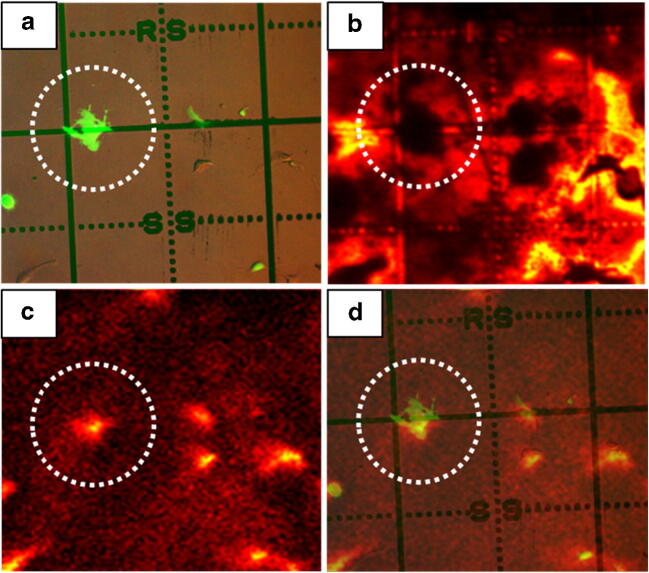
Image correlation between fluorescent microscopy images and ToF-SIMS images using grid slides. **a** Microscopy image of GFP-CCTδ transfected cell cultures on a grid slide. **b** The total ion image from the C60 analysis in positive ion mode and **c** an ion image from the GCIB analysis showing the *m/z* 184 distribution. The same cell is marked with a circle in all the images. A confirmatory overlay of the fluorescence and GCIB-SIMS image is shown in **d**. SIMS signal intensity is displayed on a thermal scale

SIMS analysis is inherently destructive in nature, so in order to maximise the signal available from the outer cell membrane, the cells were analysed first using the (CO_2_)_6k_^+^ GCIB and subsequently using C_60_^+^, both with 40 keV impact energy. The combined data sets provided a means of accurately assigning cells in the SIMS images to those in the fluorescence microscopy image and while generating the maximum intact lipid signal from the cell membrane. C_60_^+^ analysis was performed at 1.6 μm per pixel providing both a clearer outline of the cells and, importantly, an image of the underlying grid pattern and grid reference letters. Once a specific cell had been identified, the mass spectral data was extracted from the GCIB-SIMS data. Figure 2 shows a selected cell in the fluorescence image (Fig. 2 a) and the same area analysed using C_60_^+^ (Fig. 2 b). The corresponding cell in the GCIB image can be selected using a single ion image of, for example *m/z* 184. 07 (the phosphatidylcholine, PC, head group) in positive ion mode (Fig. 2 c) or *m/z* 885.5 (PI(38:4), [M-H]^−^) in negative ion mode. Figure 2 d shows an overlay of the GCIB-SIMS image of *m/z* 184.07 with the fluorescence image that has been made partially transparent, confirming the accuracy of the selection process.

To control for any effects of the transfection procedure, a mock transfection control was performed where cells had been cultured identically and treated with all the same reagents as the test cells, with the exception of the transfection plasmid itself. These mock transfection control cells are referred to as control cells. As a further control for any effects of variation between grids, such as washing efficiency, the cells from the transfected sample that did not express GFP-CCTδ and thus were not fluorescent, nor exhibited morphological changes, were included for analysis as control non-expressing cells, referred to as non-expressing cells.

As anticipated, the use of the GCIB for SIMS imaging greatly enhanced the signal levels from the intact lipid species compared to conventional, including C_60_^+^, ion beams. For the PCA analysis, the low mass signals, normally detected from cells with SIMS, were excluded from the statistical evaluation and only those above *m/z* 500 were retained. This provided a PCA result where the variation in the data was based on the intact lipids and not the intense, stable, fragment ions and substrate signals.

PCA of the positive ion mode data showed clustering of the control cells separately to the GFP-CCTδ cells and non-expressing cells when ordination plots of PC1 versus 2 were generated. However, inspection of the loadings showed no prominent negatively loading peaks suggesting that the plasmid-exposed GFP-CCTδ and non-expressing cells; particularly, the GFP-CCTδ cells produced generally more lipid signal than the control or the non-expressing cells resulting in spectra with increased lipid signal/chemical noise. A second PCA was performed with the mass range extended to include peaks down to *m/z* 350 in order to capture any potential variance in the cholesterol signal ([M + H-H_2_O]^+^ at *m/z* 369.3). The clustering pattern was unchanged and the cholesterol signal loaded positive on PC 1 suggesting that it too was relatively higher in the plasmid-exposed non-expressing and GFP-CCTδ cells compared to the control cells. PCA plots of scores and loadings for the positive ion mode data are provided in the Electronic Supplementary Material (ESM) in Fig. S1 and the cumulative variance captured by each PC in Fig. S2.

**Fig. 3:**
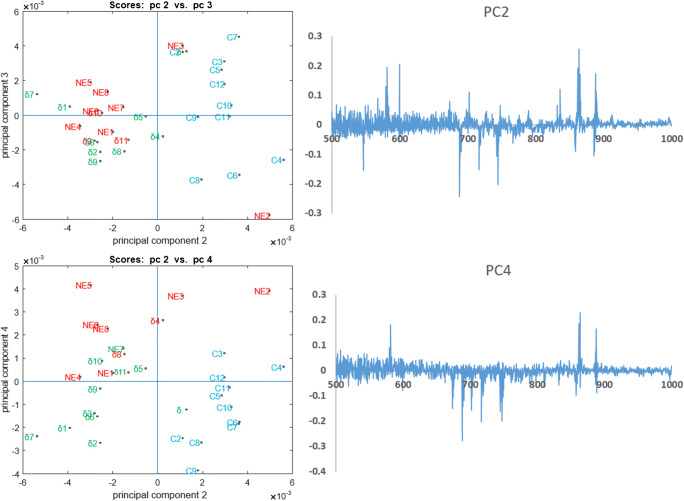
PCA result from negative ion mode GCIB-SIMS single-cell spectra. Score plot shows the scores on PC 2 versus PC 3 and PC 2 versus PC 4 with the 3 selected cell classes coloured green, red and blue for the GFP-CCTδ (δ), non-expressing (NE) and control (C) cells respectively. Loadings for PC 2 and 4 are also shown where the variable on the x-axis corresponds to *m/z*. The cumulative variance captured by each PC can be found in ESM Fig. S2

The PCA score plots of the GCIB-SIMS data in negative ion mode show good separation of the control and also the GFP-CCTδ and non-expressing cells driven by clear changes in specific lipids shown in the corresponding loading plot (Fig. 3). The positive and negative loading peaks for the 2 most descriptive principal components (PC2 and PC4) are listed in Table 1. Putative assignments generated by searching the lipid maps database using mass accuracy as the main measure of confidence are also provided and show a clear trend. The plasmid-exposed cells, especially the GFP-CCTδ cells, show a general increase in PE lipid species compared with phosphatidylinositol (PI) lipids. It should be noted that while PE lipids are commonly detected in positive ion mode in ESI-MS in SIMS analysis, intact species are normally observed as [M-H]^−^ ions. Second, the PI lipids that correlate with the GFP-CCTδ cells show a higher degree of unsaturation; control cells 1–3 double bonds, non-expressing cells 3–4 double bonds. Both the head group and the degree of saturation of the acyl chains can influence the shape of the lipid molecule and hence the physical properties of the membrane in which they are found. Lipids with small head groups (e.g. PE) are described as conical lipids and are associated with higher curvature compared with cylindrical lipids such as phosphatidylcholine (PC), and PIs with their large phosphorylated sugar head group are described as *reverse*-conical-shaped lipids. The degree of saturation alters the overall shape of the lipids and also the packing density and fluidity of a cell membrane. Straight, unsaturated chains are less bulky and so smaller relative to the head group of the lipid. Unsaturated chains are bulkier and lead to less dense packing and higher membrane fluidity. Changes in lipid saturation in cancer have also been indicated in ferroptosis [26]; however, it is unlikely that the GFP-CCTδ expression has an excessively toxic effect as the cells survive for extended periods following transfection [19].

**Table 1:** List of positive and negative loading *m/z* values from PC2 and PC4 of the PCA of negative ion mode GCIB-SIMS data. Putative assignments generated from Lipid Maps are provided with mass accuracy in ppm. PI, phosphatidylinositol; PG, phosphatidylglycerol; PA, phosphatidic acid; LPA, lysophosphatidic acid; PS, phosphatidylserine; PE, phosphatidylethanolamine; SM, sphingomyelin

Detected (*m/z*)	Lipid assignment	Intact formula	Ion	Mass acc./ppm
PC2 positive loadings				
887.556	PI 38:3	C_47_H_85_O_13_P	[M-H]^−^	10
863.56	PI 36:1	C_45_H_85_O_13_P	[M-H]^−^	2
861.551	PI 36:2	C_45_H_83_O_13_P	[M-H]^−^	2
835.536	PI 34:1	C_43_H_81_O_13_P	[M-H]^−^	3
831.489	PI 34:3	C_43_H_77_O_13_P	[M-H]^−^	15
789.539	PG 36:1	C_42_H_81_O_10_P	[M-H]^−^	4
775.546	PA O-41:6	C_44_H_77_O_7_P	[M-H]^−^	3
747.531	PI 26:3;O	C_35_H_61_O_14_P	[M-H]^−^	12
735.363	PI 26:3;O	C_39_H_75_O_8_P	[M-H]^−^	24
701.529	PA 36:1	C_37_H_73_O_8_P	[M-H]^−^	25
673.489	PA 34:1	C_37_H_71_O_8_P	[M-H]^−^	12
599.330	LPA 28:6;O	C_31_H_51_O_8_P	[M-H]^−^	12
581.318	PI-frag	C_31_H_51_O_8_P	[M-FA(20:4)]^−^	12
579.305	LPI O-18:2	C_27_H_51_O_11_P	[M-H]^−^	15
579.309	PA 28:6	C_31_H_49_O_8_P	[M-H]^−^	6
PC2 negative loadings				
888.564	PS 44:7	C_50_H_84_NO_10_P	[M-H]^−^	13
885.549	PI 38:4	C_47_H_83_O_13_P	[M-H]^−^	1
858.520	PS 42:8*	C_48_H_78_NO_10_P	[M-H]^−^	11
857.519	PI 36:4	C_45_H_79_O_13_P	[M-H]^−^	1
766.538	PE 38:4	C_43_H_78_NO_8_P	[M-H]^−^	1
744.564	PE 36:1	C_41_H_80_NO_8_P	[M-H]^−^	12
742.543	PE 36:2	C_41_H_78_NO_8_P	[M-H]^−^	6
716.534	PE 34:1	C_39_H_76_NO_8_P	[M-H]^−^	15
690.518	PE 32:0	C_37_H_74_NO_8_P	[M-H]^−^	15
687.551	SM 34:1	C_39_H_79_N_2_O_6_P	[M-CH_3_]^−^	10
PC4 positive loadings				
887.557	PI 38:3	C_47_H_85_O_13_P	[M-H]^−^	10
863.567	PI 36:1	C_45_H_85_O_13_P	[M-H]^−^	2
861.551	PI 36:2	C_45_H_83_O_13_P	[M-H]^−^	2
581.318	PA 28:5	C_31_H_51_O_8_P	[M-H]^−^	12
PC4 negative loadings				
888.564	PS 44:7	C_50_H_84_NO_10_P	[M-H]^−^	13
857.519	PI 36:4	C_45_H_79_O_13_P	[M-H]^−^	1
831.489	PI 34:3	C_43_H_77_O_13_P	[M-H]^−^	41
810.529	PS 38:4	C_44_H_78_NO_10_P	[M-H]^−^	0.2
747.531	PI 26:3;O	C_35_H_61_O_14_P	[M-H]^−^	12
744.564	PE 36:1	C_41_H_80_NO_8_P	[M-H]^−^	12
716.534	PE 34:1	C_39_H_76_NO_8_P	[M-H]^−^	15
701.530	PA 34:1	C_37_H_71_O_8_P	[M-H]^−^	12
687.551	SM 34:1	C_39_H_79_N_2_O_6_P	[M-CH_3_]^−^	10
599.330	LPI 18:0	C_27_H_53_O_12_P	[M-H]^−^	12

The changes detected upon expression of the phenotype in this study suggest an overall increase in membrane curvature from the increase in PE content and a possible increase in fluidity from the increase in unsaturated PI lipids. Ostrowski et al. have previously reported a relative enrichment of 2-AEP (an ethanolamine analogue in tetrahymena) compared with PC containing lipids at the mating junction in tetrahymena using SIMS to compare the signal from the respective head group fragment ions. Here, the GCIB allows examination of not just head group fragment ions but intact lipids providing specific information regarding overall acyl chain composition and saturation [27].

**Fig. 4:**
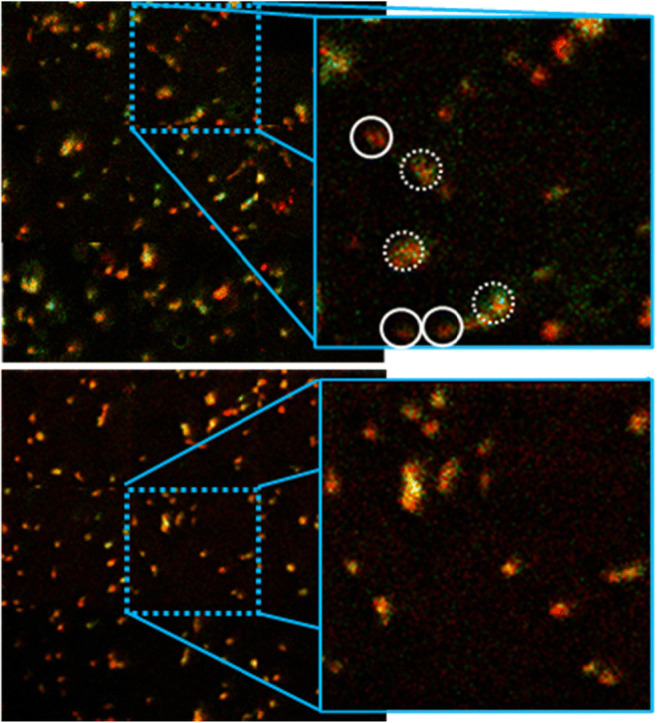
Red/green overlay SIMS images of cells imaged using the (CO_2_)_6k_^+^ GCIB. Red signal is pooled from positively loading ions from PC4 (*m/z* 863, 861, 851), and green are positive loading ions from PC4 (*m/z* 744, 716, 687). Data from the transfected cells is shown on top with cells assigned as GFP-CCTδ or non-expressing using fluorescence microscopy highlighted with dashed or solid circles respectively in the zoomed image. The same species in control cells are imaged below for comparison. Large area images are 2400 × 2400 μm^2^ and zoomed images are 800 × 800 μm^2^

In order to validate the multivariate analysis results, the strongly loading peaks were imaged from the original SIMS imaging data acquired using the (CO_2_)_6k_^+^ GCIB. Individual ion images showed the general distribution of the cells but differences between cells in the image of the plasmid-exposed (GFP-CCTδ and non-expressing) cells were not discernible. However, red/green false colour overlay images of the combined signals of several ions loading positive (PE(36:1), PE(34:1) and SM(34:1) at *m/z* 744, 716 and 687 respectively) and several ion loading negative (PI(36:2, PI(36:2 and LPI/PI-frag. at *m/z* did expose heterogeneity in the cell population. GFP-CCTδ cells were high in both green (*m/z* 744, 716, 687) and red (*m/z* 863, 861, 851) signals (so appearing yellow) with adjacent non-expressing cells appearing redder in colour (Fig. 4, top). Imaging the same ion signals in the control sample showed a much more homogenous population of cells with less overall “green” signal (Fig. 4, bottom). While verifying the PCA result, the data also underpins the power of multivariate approaches, coupled to the improved signals afforded by GCIB analysis to uncover subtle chemical changes in cell sub-populations that could not be discerned from univariate-based visual inspection (i.e. the stare and compare approach).

The increase in PE lipids in the transfected cells can be explained based on the conical shape of the PE lipid, with its relatively small head group, which facilitates the formation of highly curved structures and also results in an increase in membrane fluidity. In the case of the GFP-CCTδ transfection, it is expected that this change in lipid composition can facilitate the phenotypic changes in the cell such as the formation of tubulated membrane structures. Interestingly, increases in PE lipids are also associated with various properties of cancer cells. Increases in PE species in cancer cells have been associated with a response to stress and a way of overcoming increases in unsaturated lipids that can reduce membrane fluidity and lead to endoplasmic reticulum stress [28]. There is also a general increase in PE lipids in cancer cells and especially a migration of these to the cell membrane. The combination of these two factors has led to PEs being a potential target for therapeutic action against cancer [29]. Changes in membrane fluidity have additional implications in cancer progression. For example, Zhao et al. demonstrated connections between membrane fluidity and cell motility and found that treating breast cancer cell lines with potential anti-metastatic compounds decreased membrane fluidity [30].

## Conclusions

Here, we show that changes in CCTδ monomer levels resulting in morphological changes, including increased membrane curvature, lead to changes in lipid composition. Such alterations in lipid composition are associated with an increase in membrane fluidity and are of interest for understanding the full extent of changes that occur during the development of cancer. Specifically, PE lipids were increased in the membrane of the transfected cells.

The measurements successfully demonstrated a multimodal imaging approach using fluorescence and dual beam imaging SIMS analysis to accurately identify the cells of interest while also maximizing intact lipid signals from a single cell.

While the implementation of GCIBs for SIMS analysis has greatly improved the sensitivity of the technique to intact biomolecules, in this study, univariate analysis of the different signals was not sufficient to identify chemical changes in the transfected cells while multivariate analysis and imaging of multiple correlated peaks showed discrimination within the cellular populations based on phenotype. Further improvements to the analytical workflow may include perform the analysis on frozen hydrated samples, possibly with the implementation of water cluster ion beams that can further increase intact lipid signals, notably PEs, from single cells [31].

## Electronic supplementary material

ESM 1 (PDF 236 kb)
