# Supplementary material for: Correlated fluorescence microscopy and multi-ion beam secondary ion mass spectrometry imaging reveals phosphatidylethanolamine increases in the membrane of cancer cells over-expressing the molecular chaperone subunit CCTδ
Source: Anal Bioanal Chem. 2020 Oct 31;413(2):445–53. doi: 10.1007/s00216-020-03013-9 (PMC7806562; doi:10.1007/s00216-020-03013-9)
Supplement: Supplementary file 1 — (PDF 236 kb) [file 216_2020_3013_MOESM1_ESM.pdf]

Analytical and Bioanalytical Chemistry

Electronic Supplementary Material

Correlated fluorescence microscopy and multi-ion beam secondary ion mass spectrometry imaging reveals phosphatidylethanolamine increases in the membrane of cancer cells over-expressing the molecular chaperone subunit CCTδ

John S. Fletcher, Sanna Sämfors, Josefine Vallin, Andreas Svanström, Julie Grantham

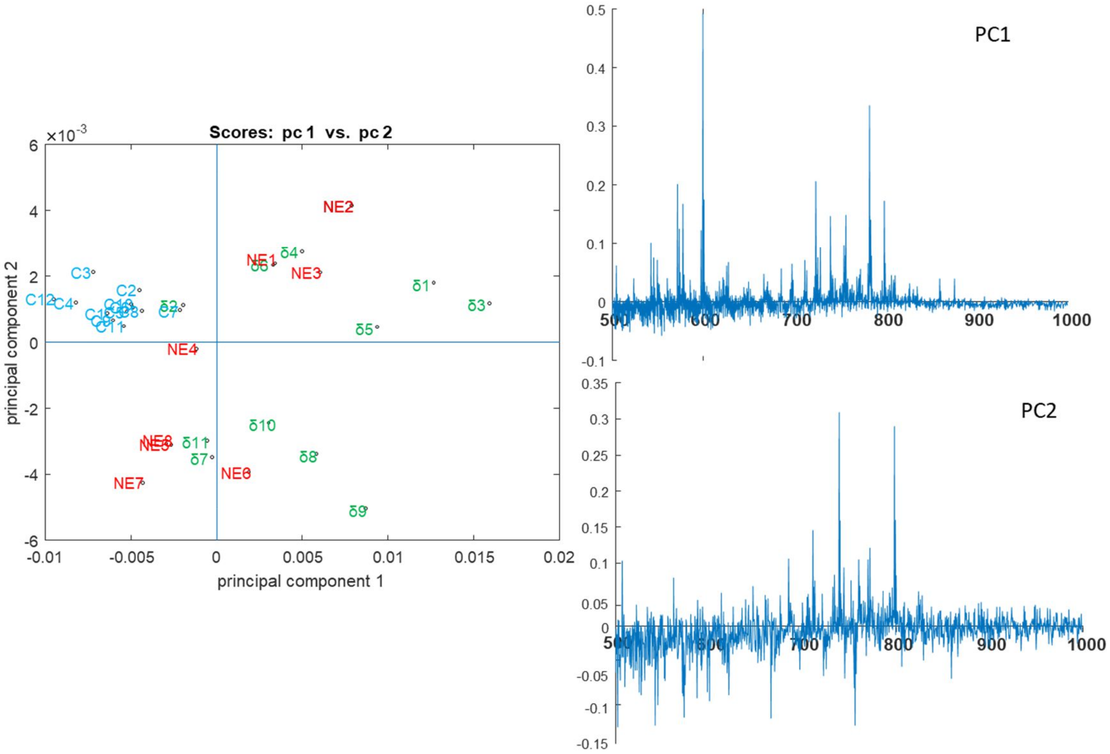

Fig. S1 PCA scores and loadings for positive ion mode data. C (blue) indicates control cells, NE (red) indicates non-expressing cells and δ (green) indicates cells showing the GFP-CCTδ phenotypic change. Loadings for PC 1 and 2 are also shown where the variable on the x -axis corresponds to m/z

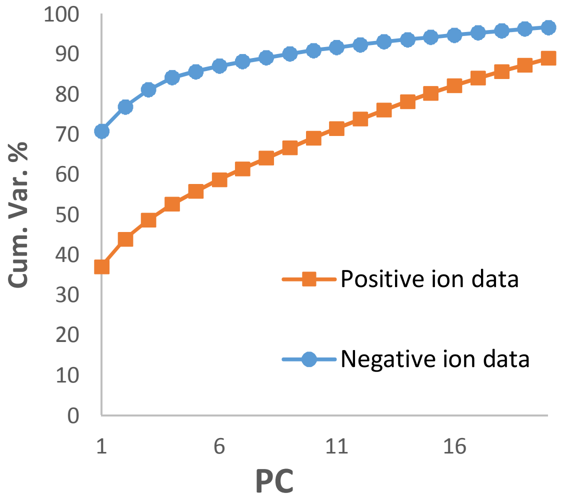

Fig. S2 Cumulative variance capture in the PCA of positive (orange) and negative (blue) data
